# Transcriptomic Studies Reveal that the *Rhizobium leguminosarum* Serine/Threonine Protein Phosphatase PssZ has a Role in the Synthesis of Cell-Surface Components, Nutrient Utilization, and Other Cellular Processes

**DOI:** 10.3390/ijms20122905

**Published:** 2019-06-14

**Authors:** Paulina Lipa, José-María Vinardell, Monika Janczarek

**Affiliations:** 1 Department of Genetics and Microbiology, Institute of Microbiology and Biotechnology, Faculty of Biology and Biotechnology, Maria Curie-Skłodowska University, Akademicka 19 St., 20-033 Lublin, Poland; paulina.lipa56@gmail.com; 2 Department of Microbiology, Faculty of Biology, University of Sevilla, Avda. Reina Mercedes 6, 41012 Sevilla, Spain; jvinar@us.es

**Keywords:** *Rhizobium leguminosarum*, serine/threonine protein kinases, serine/threonine protein phosphatases, transcriptomics, gene expression, surface polysaccharides, exopolysaccharide, symbiosis, clover, nitrogen fixation

## Abstract

*Rhizobium leguminosarum* bv. *trifolii* is a soil bacterium capable of establishing symbiotic associations with clover plants (*Trifolium* spp.). Surface polysaccharides, transport systems, and extracellular components synthesized by this bacterium are required for both the adaptation to changing environmental conditions and successful infection of host plant roots. The *pssZ* gene located in the Pss-I region, which is involved in the synthesis of extracellular polysaccharide, encodes a protein belonging to the group of serine/threonine protein phosphatases. In this study, a comparative transcriptomic analysis of *R. leguminosarum* bv. *trifolii* wild-type strain Rt24.2 and its derivative Rt297 carrying a *pssZ* mutation was performed. RNA-Seq data identified a large number of genes differentially expressed in these two backgrounds. Transcriptome profiling of the *pssZ* mutant revealed a role of the PssZ protein in several cellular processes, including cell signalling, transcription regulation, synthesis of cell-surface polysaccharides and components, and bacterial metabolism. In addition, we show that inactivation of *pssZ* affects the rhizobial ability to grow in the presence of different sugars and at various temperatures, as well as the production of different surface polysaccharides. In conclusion, our results identified a set of genes whose expression was affected by PssZ and confirmed the important role of this protein in the rhizobial regulatory network.

## 1. Introduction

The natural environment is a valuable reservoir of many microorganisms. One of such reservoirs is soil, which can be inhabited by extremely high numbers of diverse microorganisms (from 4,000 to 50,000 different microorganisms and up to 10^10^ bacterial cells in 1 g of soil) [1]. One of the important groups of these soil microorganisms is nitrogen-fixing symbiotic bacteria belonging to the family *Rhizobiaceae*, which are collectively called rhizobia [2,3]. These heterotrophic microorganisms possess extremely large genomes (up to 9 Mbp) and show a very high metabolic plasticity, thanks to which they can exist in two lifestyles, as free-living bacteria and as endosymbionts of legume plants [4,5]. Rhizobia participate in the biological fixation of atmospheric dinitrogen in associations with their compatible host plants, supplying ~200 million tons of this element per year to the global nitrogen cycle; that is, almost a half of nitrogen compounds introduced to the environment as artificial fertilizers [6,7,8]. Thus, this type of plant–microbe interaction plays a crucial role in the functioning of the biosphere since it increases soil fertility and field crops.

Nitrogen-fixing symbiosis is a multi-step process which requires coordination between the macro- and the microsymbiont and involves an exchange of signals between the compatible partners. These signal molecules include plant flavonoids and bacterial lipochitooligosaccharides (Nod factors) and surface polysaccharides (PSs); among the latter, exopolysaccharide (EPS) and lipopolysaccharide (LPS) are the most important [3,9,10,11]. Recently, a signal role of low-molecular-weight (LMW) EPS in early stages of symbiosis (i.e., host root infection) has been confirmed, and a plant receptor responsible for the recognition of this PS has been identified [12,13]. However, before rhizobia find compatible host plants, they have to survive in soil as free-living bacteria and are exposed to various environmental factors such as nutrient limitations, drought, salinity, temperature changes, the presence of heavy metals, and oxidative stress [14,15,16,17,18,19,20]. Therefore, rhizobia have developed several features and strategies that allow them to adapt to these conditions. One of these adaptations is a complex composition of their bacterial envelope, in which various PSs have been identified. These rhizobial PSs include LPS, EPS, capsular polysaccharide (CPS), as well as two PSs recently characterized in *Rhizobium leguminosarum*: neutral (NP, glucomannan) and gel-forming (GPS) polysaccharides [3,9,21]. CPS is tightly associated with the rhizobial surface and its structure in most species is very similar or even identical to that of EPS. In contrast, EPS is weakly associated with the bacterial surface and is released in large amounts to the environment. Furthermore, EPS, which forms the most external layer of the rhizobial cells, plays an important protective role against desiccation, nutrient limitation, and other stress conditions occurring in the soil. Cyclic β-glucans (CGs), which are located in the periplasmic space, are involved in bacterial adaptation to hypo-osmotic conditions. All these PSs are required for different stages of symbiosis, such as attachment to and biofilm formation on plant roots, as well as for the successful infection of legumes and adaptation to conditions prevailing inside nodules, i.e., specific organs formed by legume roots, in which rhizobia are hosted [22,23,24,25,26]. It has been established that EPS is especially important in symbioses with legumes that form indeterminate-type nodules (e.g., clovers with *R. leguminosarum* bv. *trifolii,* vetch and peas with *R. leguminosarum* bv. *viciae,* and alfalfa with *Sinorhizobium meliloti*), where this PS is involved in the initiation and propagation of tubular structures inside host roots, called infection threads (IT) [27]. However, some exceptions are known (e.g., EPS of *S. fredii* HH103 is not required for the nodulation of *Glycyrrhiza uralensis,* which also forms indeterminate-type nodules) [28]. EPS is a major component of the IT matrix and is involved in the suppression of plant defense responses [29,30]. The significant role of EPS in the rhizobial adaptation to both soil conditions and symbiosis with legumes has been confirmed by phenotypes of various EPS-deficient mutant strains (e.g., *R. leguminosarum* bvs. *trifolii* and *viciae*, and *S. meliloti*), which were inefficient in host root infection and nitrogen fixation [27,31,32,33].

Due to the very important role of EPS, the synthesis of this polymer had become an object of many studies in several rhizobial representatives. However, despite these numerous studies and the determination of the chemical structures of EPS for several rhizobial species, the biosynthetic pathways and regulation of the production of this PS are known only fragmentarily [34,35]. The EPS of *R. leguminosarum* is composed of octasaccharide subunits, which contain D-glucose, D-glucuronic acid, and D-galactose residues in a molar ratio 5:2:1 and are substituted with the O-acetyl and pyruvyl groups [36,37,38,39]. This PS is synthesized by a multi-enzymatic complex located in the bacterial inner membrane. To date, only the function of a few proteins involved in EPS synthesis has been established experimentally in this bacterium. PssA, PssDE, PssC, and PssS glycosyl transferases are engaged in the first four steps, whereas PssJ is probably involved in the last step of the EPS subunit assembly [40,41,42,43,44,45]. PssM encodes a ketal pyruvate transferase responsible for the pyruvylation of the EPS subunit [46], and proteins PssT, PssN, PssL, PssP, and PssP2 are components of the EPS polymerization and export system [47,48,49,50]. A great majority of these proteins (with the exception of PssA and PssL) are encoded by genes located in a large chromosomal cluster called Pss-I [45,51,52]. Mutations in *pssA, pssD, pssE*, and *pssS* genes totally abolish EPS synthesis in *R. leguminosarum* and, in consequence, the effective symbiosis with its host plants [31,41,45,53].

EPS synthesis in *R. leguminosarum* is regulated by several proteins (PsiA, PsrA, ExoR, and RosR) and environmental factors (phosphate and nitrogen limitations, carbon source, flavonoids) [43,54,55,56,57,58]. Among these proteins, PsiA, PsrA, and ExoR negatively affect EPS synthesis, whereas RosR positively regulates this process.

Furthermore, as previously reported, the *pssZ* gene, which is located in the Pss-I region, is also involved in EPS synthesis [59]. In-silico sequence analysis showed that this gene encodes a protein belonging to the family of serine/threonine protein phosphatases (STPs), which are involved in regulation of various cellular processes in bacteria, including growth and division, motility, envelope biogenesis, biofilm formation, cell aggregation, regulation of transcription and translation, and signaling [60,61,62,63]. Until now, most STPs have been characterized in Gram-positive bacteria, and only a few examples of these enzymes in Gram-negative bacteria have been reported. The PssZ protein is the first STP described in *Rhizobiaceae* representatives to date, as well as the first case of linking this type of enzymatic activity with bacterial EPS synthesis pathways. Studies performed by our research group have shown that a mutation in this gene had pleiotropic effects and significantly affected several cellular processes [59]. A *pssZ* mutant of *R. leguminosarum* bv. *trifolii* Rt297 exhibited several physiological and symbiotic defects, among them, the lack of EPS synthesis and decreased growth and cell motility. The inhibition of EPS production was correlated with a reduced ability to form biofilms and a dramatic decrease in the symbiotic effectiveness with red clover (*Trifolium pratense*), resulting in the formation of deformed root nodules, which were inefficient in nitrogen fixation [59]. These data indicated that the PssZ protein is not only indispensable for EPS biosynthesis, but also required for the proper functioning of *R. leguminosarum* bv. *trifolii* cells in symbiosis.

Reversible phosphorylation is a key mechanism that regulates several cellular processes in both prokaryotes and eukaryotes. Many recent studies indicate that regulatory pathways controlled by Hanks-type serine/threonine kinases (STKs) and serine/threonine phosphatases (STPs) play an important role in the regulation of many bacterial processes, including growth and cell division, cell wall biogenesis, sporulation, biofilm formation, stress response, metabolic and developmental processes, and interactions of both pathogenic and symbiotic bacteria with their hosts [60,61,62,63]. STKs and STPs are not DNA-binding proteins; therefore, they exert a regulatory role via post-translational modifications of their protein targets, among them, several regulatory proteins of other signalling cascades.

In this work, we performed a comparative transcriptomic analysis of *R. leguminosarum* bv. *trifolii* wild-type Rt24.2 and its derivative, *pssZ* mutant Rt297. This analysis provided evidence on the PssZ-mediated regulation of gene expression in this bacterium. It was established that PssZ influenced the expression of a large group of genes involved in many processes such as transcription and translation, the synthesis of cell-surface components and polysaccharides, motility, and different metabolic pathways. Our results suggest that PssZ plays an important role in the regulation of various cellular processes in *R. leguminosarum* bv. *trifolii*.

## 2. Results

### 2.1. RNA-Seq Analysis of the Wild-Type Rt24.2 and pssZ Mutant Rt297 Strains

In a previous study, we showed that the *pssZ* mutation causes pleiotropic effects in rhizobial cells, including the lack of EPS production, reduced growth kinetics and motility, and failure in host root infection [59]. These findings suggest that *pssZ* might play a broad regulative function in *R. leguminosarum* bv. *trifolii*. Therefore, in the present study, we have performed a comparative transcriptomic analysis of the wild-type Rt24.2 and its derivative, *pssZ* mutant strain Rt297, to establish a set of genes differentially expressed in these two strains. Cultures of bacteria grown in the rich 79CA medium up to the middle exponential growth phase when bacterial cells intensively divide were used for total RNA isolation. For these analyses, a draft genome sequence of Rt24.2 which was obtained by us earlier was used as a reference strain (181 contigs with a total length of 7,653,217 bp, in which 7,374 putative coding regions were identified) [58,64].

**Figure 1:**
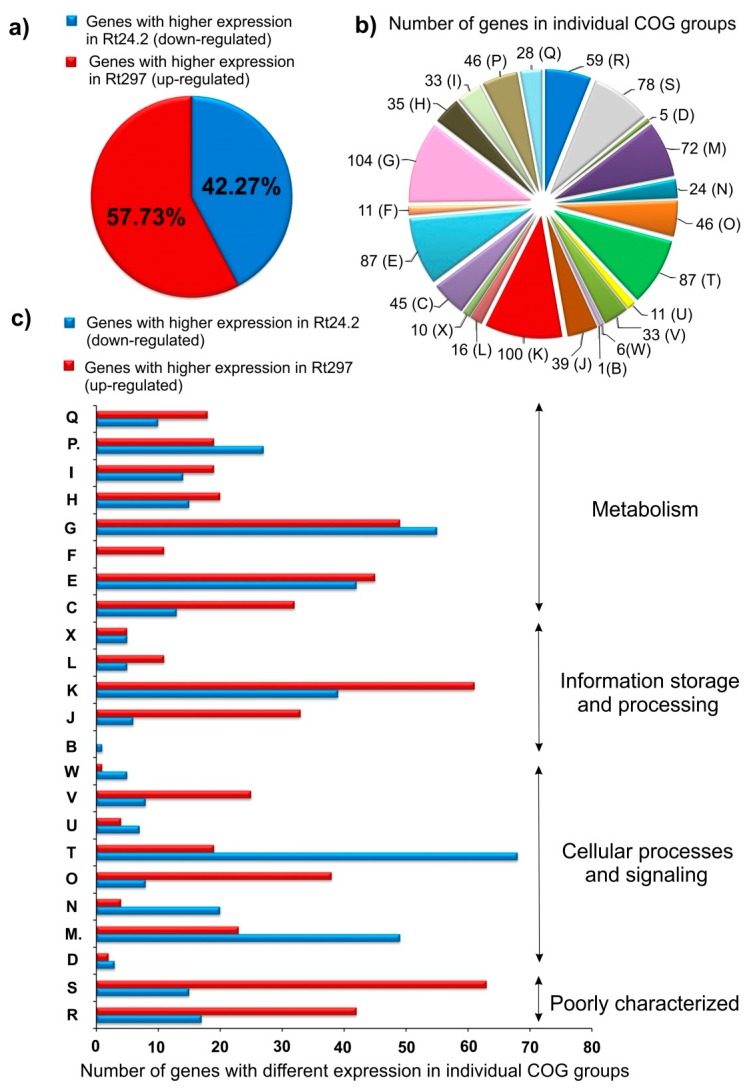
The genes differentially expressed in the *pssZ* mutant Rt297 in relation to the wild-type strain *R. leguminosarum* bv. *trifolii* Rt24.2. (**a**) Global classification of the genes into up-regulated ones (red color), whose expression was higher, and down-regulated ones (blue color), whose expression was lower in the *pssZ* mutant than in the wild-type background, respectively; (**b**) Numbers of genes from the individual functional groups (COGs M-S) differentially expressed in the Rt24.2 and Rt297 strains; (**c**) the number of genes from individual COGs differentially expressed in the Rt24.2 and Rt297 strains (up- and down-regulated genes in the *pssZ* mutant); genes encoding hypothetical proteins, which were not classified to COGs, constituted 14.08%. Abbreviations of COGs: B = Chromatin structure and dynamics, C = Energy production and conversion, D = Cell cycle control, cell division, chromosome partitioning, E = Amino acid transport and metabolism, F = Nucleotide transport and metabolism, G = Carbohydrate transport and metabolism, H = Coenzyme transport and metabolism, I = Lipid transport and metabolism, J = Translation, ribosomal structure and biogenesis, K = Transcription, L = Replication, recombination and repair, M = Cell wall/membrane/envelope biogenesis, N = Cell motility, O = Post-translational modification, protein turnover, and chaperones, P = Inorganic ion transport and metabolism, Q = Secondary metabolites biosynthesis, transport, and catabolism, R = General function prediction only, X = Mobilom, S = Function unknown, T = Signal transduction mechanisms, U = Intracellular trafficking, secretion, and vesicular transport, V =Defense mechanisms, W = Extracellular structures.

To compare gene expression profiles in Rt24.2 and Rt297, three cDNA libraries for each strain were prepared and sequenced as pair-end reads using Illumina MiSeq with SBS technology. After filtering off primer-adaptor sequences and low-quality reads, the remaining reads were mapped to the reference Rt24.2 genome in order to identify differentially expressed genes (DEGs) in the Rt24.2 and Rt297 strains. An analysis of the functional composition of the wild-type strain transcriptome showed that the most numerously represented categories were those related to metabolic processes, especially functional groups (COGs) involved in the uptake and metabolism of carbohydrates (COG G), amino acids (E), and inorganic ions (P), as well as an energy production and conversion (C) (Figure 1, Supplementary Table S1). Furthermore, large numbers of genes related with transcription (K) and translation processes (J), cell envelope biogenesis (M), and poorly characterized genes (classes R and S), were highly represented in the *R. leguminosarum* transcriptome. Based on fold changes of gene expression in the wild type and the *pssZ* mutant (log_2_ Rt24.2/Rt297 values >1.4), we established that 996 genes were transcribed at significantly different levels in these two strains. These data indicate that PssZ is engaged in the regulation of the expression of a large group of rhizobial genes, suggesting that this protein plays an important role in Rt24.2 regulatory networks (Supplementary Table S1). Among these DEGs, slightly more genes were up-regulated (57.73%), whereas 42.27% were down-regulated in the *pssZ* mutant (Figure 1A). Among the 996 genes analyzed, 83.94% were successfully classified into particular COGs (Figure 1B) [65]. Most of these genes belonged to the following functional groups: transport and metabolism of carbohydrates (COG G) (9.15%) and amino acids (E) (7.66%), transcription (K) (8.80%), signal transduction (T) (7.66%), and cell wall/membrane/envelope biogenesis (M) (6.34%). Many DEGs were also classified to COGs encompassing poorly characterized proteins with general (R) (5.20%) and unknown functions (S) (6.86%) (Figure 1B). Moreover, when individual COGs were analyzed, we have found that in the case of COGs involved in signaling and several cellular processes, a high number of genes were down-regulated in the *pssZ* mutant in relation to the wild type (i.e., signal transduction (T), cell wall/membrane/envelope biogenesis (M), cell motility (N), extracellular structures (W) and intracellular trafficking, secretion and vesicular transport (U)) (Figure 1C). In contrast, a great majority of genes belonging to COGs involved in information storage and processing (J, K, L, O), cell metabolism (C, F, H, I, Q), and those from the COGs R and S were up-regulated in Rt297.

With respect to individual DEGs, nearly 12% of the PssZ regulon exhibited more than 32-fold changed expression between the wild type and the *pssZ* mutant (log_2_ fold change 24.2/297 >5 or <−5) (Supplementary Table S2). This set of genes showing very high differential expression includes *Rt659_14* encoding a sugar ABC transporter permease (log_2_ 24.2/297 = −10.16), *Rt659_15* encoding a sugar-binding protein (−11.12), *Rt659_20* encoding a glycine/betaine ABC transporter (−11.02), *Rt651_2* encoding a cold-shock protein (−12.65), *Rt651_33* encoding a LuxR family transcriptional regulator (−12.65), *Rt659_32* encoding a LysR family transcriptional regulator (−10.87), and genes encoding glycosyl transferases involved in EPS synthesis (*Rt772_9*= 13.09, *Rt772_10* = 13.07, and *Rt772_14* = 12.59).

#### 2.1.1. Transcription, Translation, and Signal Transduction Mechanisms

**Figure 2:**
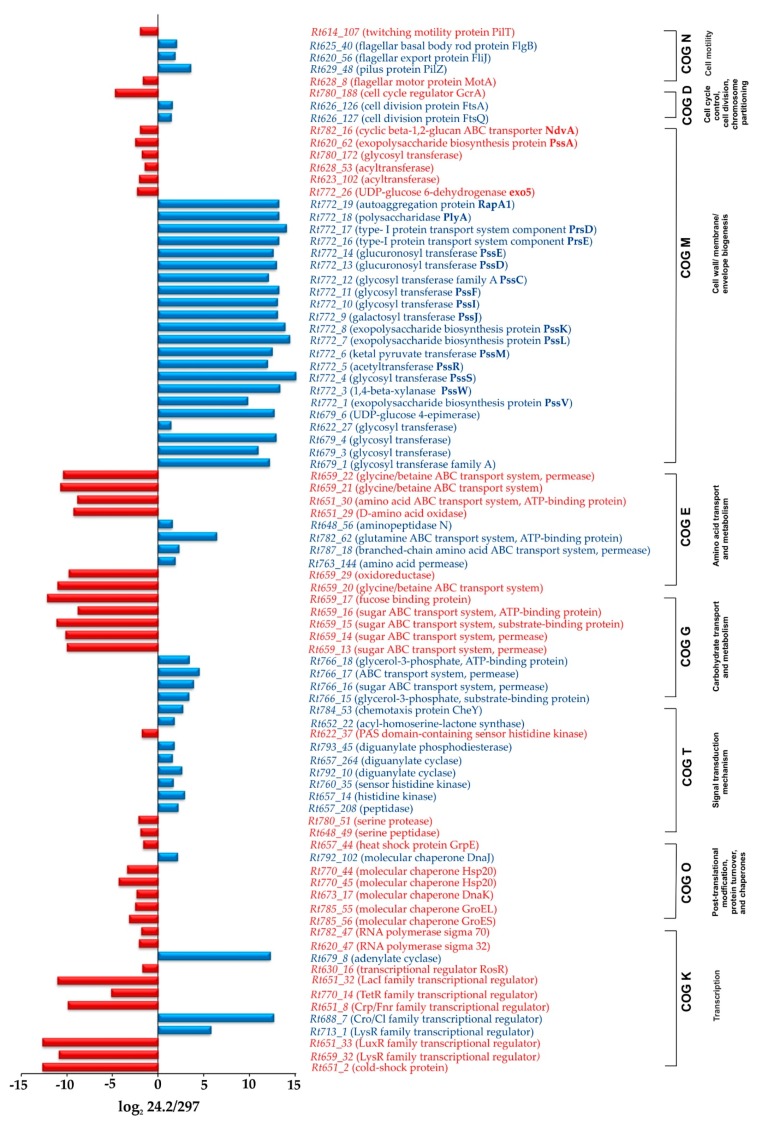
The representative genes from the individual COGs differentially expressed in the *pssZ* mutant Rt297 in relation to the wild-type strain Rt24.2. Functions of putative proteins encoded by these genes are given in brackets.

A functional category that is highly represented in the PssZ regulon (100 genes) is transcription (COG K) (Figure 1B,C). A majority of the genes from this COG were up-regulated (61 genes), whereas 39 genes were down-regulated in the *pssZ* mutant. These DEGs encoded many proteins belonging to various transcriptional regulatory families such as LysR, LuxR, Crp/Fnr, LacI, RpiR, AraC, and TetR (Table S1, Figure 2); e.g., Rt659_32 and Rt713_1 (LysR family), Rt688_7 (Cro/Cl family), Rt651_33 (LuxR family), Rt651_8 (Crp/Fnr family), and Rt770_14 (TetR family). A catabolic protein Crp/Fnr (Rt651_8), a regulatory LacI-type protein (Rt651_32), Rt619_151 (ROK), and an adenylate cyclase Rt679_8 are most probably engaged in the regulation of carbon metabolism (Figure 2). Moreover, genes *Rt782_65, Rt766_60, Rt627_60,* and *Rt764_21,* encoding regulators from the GntR family and probably engaged in general metabolism, and *Rt793_203* and *Rt793_293,* encoding OmpR-type transcription factors, were down-regulated in the *pssZ* mutant. Genes *Rt620_47* and *Rt782_47,* encoding RNA polymerase sigma subunits σ^32^ and σ^70^, respectively, were overexpressed in the Rt297 mutant.

Additionally, several genes associated with translation and post-translational modifications (COGs J and O) were expressed at different levels in these two strains. Many genes encoding ribosomal proteins of both 50S and 30S subunits were identified as DEGs, and a majority of them were up-regulated in the *pssZ* mutant (e.g., *Rt775_6, Rt775_7, Rt775_8*, *Rt775_13,* and *Rt775_14)*. These data suggest the occurrence of some disturbances in ribosome biogenesis and/or in the translation process in this strain.

Several genes classified into the COG O, which encode putative chaperons, a heat shock protein, and proteases, were expressed at higher levels in the *pssZ* mutant than in the wild type (e.g., Rt785_56 (GroES), Rt785_55 (GroEL), Rt673_17 (DnaK), Rt770_45 and Rt770_44 (Hsp20), a heat shock protein Rt657_44 (GrpE), a serine protease, and peptidases (Rt648_49, Rt780_51, Rt657_208)) (Figure 2). In contrast, *Rt792_102* encoding a chaperone DnaJ was down-regulated in the mutant.

Furthermore, many genes from the COG T, which is involved in signal transduction mechanisms, were also found to belong to the PssZ regulon. A great majority of them (78.16%) were down-regulated in the *pssZ* mutant (Figure 1B,C, Table S1). Among DEGs from this functional group, several genes coding for putative sensor histidine kinases (*Rt657_14* and *Rt760_35*), a di-guanylate phosphodiesterase (*Rt793_45*), a PAS sensor protein (*Rt622_37*), a putative acyl-homoserine lactone synthase (*Rt652_22*) involved in quorum sensing, and a CheY-type chemotaxis protein (*Rt784_53*) were found (Figure 2). Interestingly, several genes encoding putative di-guanylate cyclases (e.g., *Rt657_264, Rt792_10, Rt615_41, Rt618_35, Rt620_42, Rt620_87,* and *Rt623_10*) were down-regulated in the *pssZ* mutant (log_2_ fold change 24.2/297 from 1.51 to 3.07) (Table S1). These proteins are probably engaged in the synthesis of a cyclic di-guanylate monophosphate (c-di-GMP), which is an important signal molecule involved in the regulation of many cellular processes in bacteria [66,67,68,69,70,71].

#### 2.1.2. Carbon and Amino Acid Transport and Metabolism

Besides the COGs K and T, a large part of the PssZ regulon was constituted by DEGs related to bacterial metabolism (Figure 1B,C). Among these genes, the highest numbers were those grouped in COGs G (104 genes), E (87 genes), and P (46 genes). In these COGs, similar numbers of genes were up- and down-regulated in the *pssZ* mutant. These DEGs encoded components of various transport systems and enzymes involved in the metabolism of different carbon, nitrogen, and inorganic sources. Some genes from the COG G, encoding different components of a putative sugar transport system were down-regulated in the *pssZ* mutant (*Rt766_15, Rt766_16, Rt766_17,* and *Rt766_18*), whereas other genes, encoding components of another sugar transport system, were up-regulated in this strain (*Rt659_13, Rt659_14, Rt659_15, Rt659_16,* and *Rt659_17*) (Table S1). In addition, several other genes related to the carbon metabolism were expressed at lower levels in the *pssZ* mutant than in the wild type (e.g., *Rt659_20* and *Rt659_29*) (Figure 2, Table S1).

The COG E related to nitrogen transport and metabolism encompassed a large part of the PssZ regulon as well (87 genes). Among these DEGs, genes encoding an amino acid permease (*Rt763_144*), a branched-chain amino acid ABC transporter permease (*Rt787_18*), a putative glutamine ABC transporter ATP-binding protein (*Rt782_62*), and an aminopeptidase N (*Rt648_56*) were expressed at lower levels in the mutant in relation to the wild type background. In contrast, genes coding for an amino acid oxidase (*Rt651_29),* an ATP-binding protein of an amino acid ABC-type transport system (*Rt651_30*), and components of a putative glycine/betaine transport system (*Rt659_21* and *Rt659_22*) were up-regulated in Rt297.

In summary, the large number of DEGs found in the COGs G and E suggests the occurrence of some disturbances in metabolic pathways in cells of the *pssZ* mutant.

#### 2.1.3. Synthesis of Cell-Surface Components

Many DEGs associated with cell envelope biogenesis and the synthesis of different PSs were also identified in the PssZ regulon (COG M) (Figure 1B,C). A significant majority of them (68.06%) were down-regulated in the *pssZ* mutant. Among these DEGs, a large number of genes involved in the synthesis of sugar precursors (*Rt679_6, Rt772_26)* and different PSs were found (e.g., *Rt772_1, Rt772_4, Rt772_13, Rt679_1, Rt679_3, Rt679_4, Rt622_27*) (Figure 2). Some of these genes are located in the Pss-I region and are engaged in EPS synthesis (*Rt772_4, Rt772_9, Rt772_10, Rt772_11, Rt772_12, Rt772_13,* and *Rt772_14,* encoding glycosyl transferases, *Rt772_5, Rt772_6* and *Rt772_8* encoding enzymes adding non-sugar modification to EPS subunits, and *Rt772_7* encoding PssL engaged in EPS export) (Figure 2). These genes were strongly down-regulated in the *pssZ* mutant (log_2_ fold change 24.2/297 from 9.79 to 15.10). Some other genes located in the Pss-I region, such as *Rt772_18* and *Rt772_19,* which codes for polysaccharidase PlyA and autoaggregation protein RapA1, are also down-regulated in Rt297. Similarly, *Rt623_91* encoding an UDP-phosphate glucose phosphotransferase, which is a homolog of the *R. leguminosarum* bv. *viciae* 3841 *gmsA* gene involved in NP synthesis, was slightly down-regulated in the *pssZ* mutant (log_2_ fold change 24.2/297 1.80).

In contrast, several genes from this COG were up-regulated in the mutant. These include *Rt772_26*(*exo5*) encoding a UDP-glucose 6-dehydrogenase, *Rt623_102* and *Rt628_53* (acyltransferases), *Rt780_172* (a putative glycosyl transferase), *Rt620_62* (glucosyl transferase PssA involved in the first step of EPS synthesis), and *Rt630_16* (a positive regulator of EPS synthesis, RosR), which showed log_2_ fold changes from −1.46 to −2.50 (Figure 2). The expression of *Rt782_16*, encoding an ABC transporter of CG (NdvA) was also up-regulated in the *pssZ* mutant.

#### 2.1.4. Genes Involved in Cell Cycle and Motility

A few DEGs related to the regulation of the bacterial cell cycle were identified in the PssZ regulon. Among these genes, *Rt626_126* and *Rt626_127*, encoding cell division proteins FtsA and FtsQ, were down-regulated in the *pssZ* mutant (log_2_ fold change 1.54 and 1.43, respectively) (Figure 2). In contrast, *Rt780_188*, which codes for a cell cycle regulator GcrA, was up-regulated in this strain (log_2_ fold change −4.72). These data are in congruence with our earlier observation that the *pssZ* mutant grew significantly slower and had a longer generation time than the wild-type strain [59].

Moreover, several DEGs associated with the formation and/or functioning of pilus and flagellar structures required for cell motility were identified in the PssZ regulon. For example, *Rt629_48, Rt620_56, Rt793_203*, and *Rt625_40* were down-regulated, whereas *Rt628_8, Rt780_212,* and *Rt614_107* were up-regulated in the mutant strain. These results suggest some disturbances in the functioning of these cell-surface structures and confirm our previous findings that the mutant cells were characterized by significantly slower swarming motility in comparison to wild-type cells [59].

#### 2.1.5. Analysis of Transcriptional Fusions in Rt297 and Rt24.2 Strains

**Figure 3:**
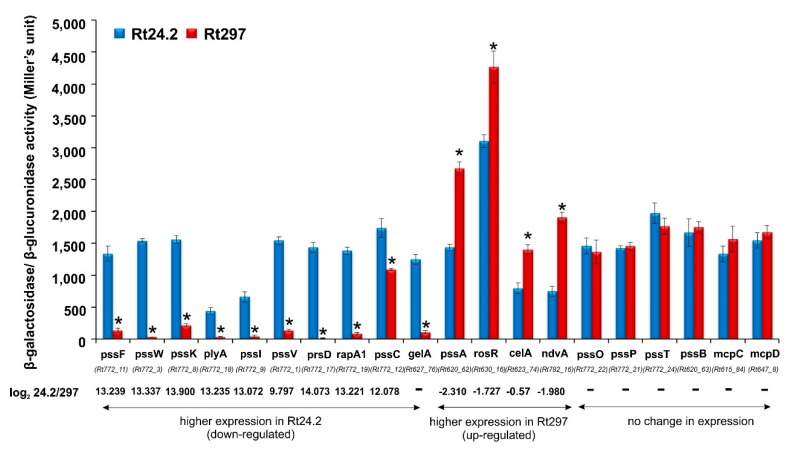
The transcriptional activity of rhizobial promoters in the wild-type Rt24.2 and the *pssZ* mutant strains determined in β-galactosidase or β-glucuronidase activity assays and presented as Miller units. Significant differences in the transcriptional activity of individual promoters between Rt24.2 and Rt297 strains are marked with * (*p* < 0.05, one-way Anova). The log_2_ fold change 24.2/297 values for individual genes obtained in RNA-Seq analysis is given below the diagram; genes, for which differences in expression between Rt24.2 and Rt297 in RNA-Seq were not found, are marked with “-“.

To validate the data obtained from the RNA-Seq analyses, several genes representative of the PssZ regulon, for which different expression between the wild-type and the *pssZ* mutant was observed, as well as genes not belonging to this regulon, whose expression was not affected by PssZ, were chosen. The transcriptional activity of the genes from these two groups was determined using fusion plasmids containing promoter regions of these genes subcloned upstream of promoterless *lacZ* or *gusA* reporter genes. These plasmids were introduced into both the Rt24.2 and Rt297 strains by bi-parental conjugation, and β-galactosidase/β-glucuronidase activity assays were performed. The genes chosen for the transcriptional analysis exhibited a wide range of expression levels, as it was determined in the wild-type background (values from 3102 for *rosR-lacZ* to 438 Miller units for *plyA-lacZ*) (Figure 3).

When the transcriptional activity of the individual gene studied was compared between the wild type and the *pssZ* mutant backgrounds, significant differences in the expression levels were found for those genes, in which differences in expression assessed by the RNA-Seq analysis were also found. Higher expression levels in Rt24.2 in comparison to Rt297 were determined for the following genes: *Rt772_11(pssF), Rt772_3(pssW), Rt772_8(pssK), Rt772_18(plyA), Rt772_9(pssI), Rt772_1(pssV), Rt772_18(rapA1),* and *Rt772_12(pssC),* whereas lower expression was established for *Rt620_62(pssA), Rt782_16(ndvA),* and *Rt630_16(rosR)*. Furthermore, based on the β-galactosidase activity assay, the transcriptional activity of genes that are not members of the PssZ regulon (based on the RNA-Seq analysis) was on similar levels in both strains Rt24.2 and Rt297 (e.g., *pssO, pssN, pssT, pssP, pssB, mcpC,* and *mcpD*) (Figure 3). Thus, these results confirmed that PssZ is involved in the regulation of the expression of several genes associated with the synthesis of various rhizobial PSs and other surface components.

In summary, the results obtained from the β-galactosidase/β-glucuronidase activity assays are in congruence with those obtained from the RNA-Seq analysis, thus confirming the reliability of the transcriptomic analysis of the *R. leguminosarum* PssZ regulon described in this work.

### 2.2. Phenotypic Characteristics of the Wild-Type Strain Rt24.2 and Its Derivatives

In order to confirm the involvement of the *pssZ* gene in several cellular processes, as suggested by the transcriptomic data obtained for the *pssZ* mutant and the wild type, we determined some phenotypic traits of these strains. In addition, a complemented version of the *pssZ* mutant, Rt297(pPL1), as well as a *pssZ-*overexpressing strain, Rt24.2(pPL1), were included in these experiments.

#### 2.2.1. Growth at a Wide Range of Temperatures

**Figure 4:**
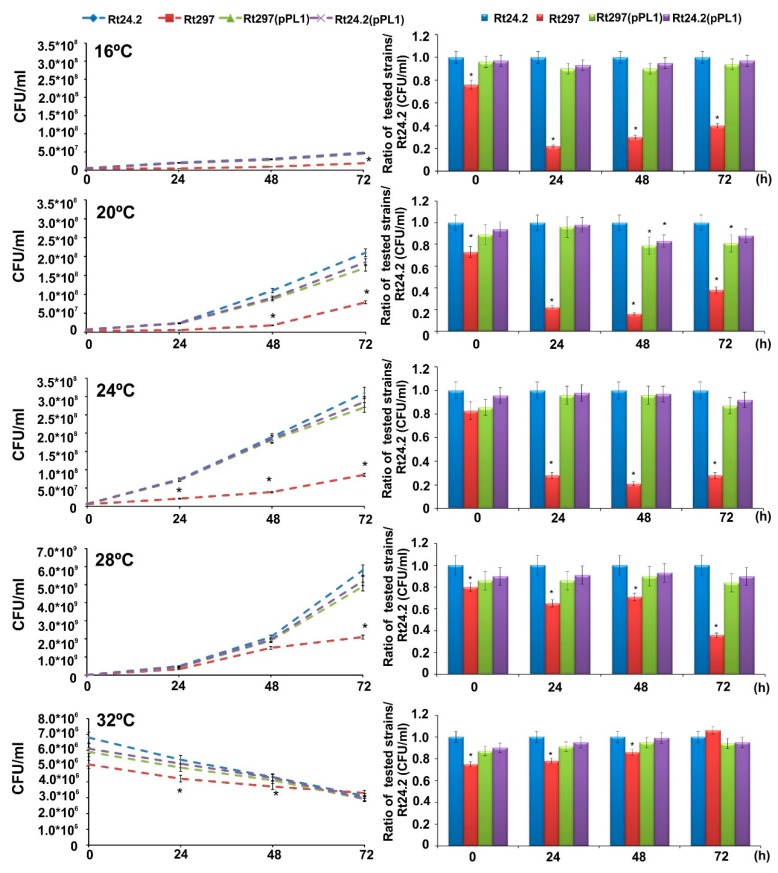
The growth kinetics of the wild-type strain Rt24.2 and its derivatives Rt297, Rt297(pPL1), and Rt24.2(pPL1) at various temperatures, presented as culture CFU/mL values (left panel) and as a ratio of a CFU/mL value of the individual strain tested per a CFU/mL value of Rt24.2, determined for each particular temperature and time point tested (right panel). Significant differences between Rt24.2 and its derivatives are marked with * (*p* < 0.05, one-way ANOVA).

The growth kinetics of the Rt297, Rt24.2, Rt297(pPL1), and Rt24.2(pPL1) strains at 16, 20, 24, 28, and 32 °C during 72 h was determined in 79CA medium containing 1% glycerol (*w*/*v*) as a carbon source. To avoid the influence of the EPS produced by the individual strains, the CFU/mL parameter was used instead of the culture optical density. The optimal growth temperature for all strains was 28 °C, whereas, also in all cases, 16 °C and 32 °C provoked slight growth and loss of viability, respectively (Figure 4).

Moreover, the *pssZ* mutant was characterized by a significantly slower growth in relation to the remaining strains at all tested temperatures (with the exception of 32 °C), that was in congruence with the previous results of our group obtained at 28 °C [59]. However, this mutant proved to be slightly more resistant to 32 °C than the rest of the strains tested.

#### 2.2.2. Utilization of Different Sugars

**Figure 5:**
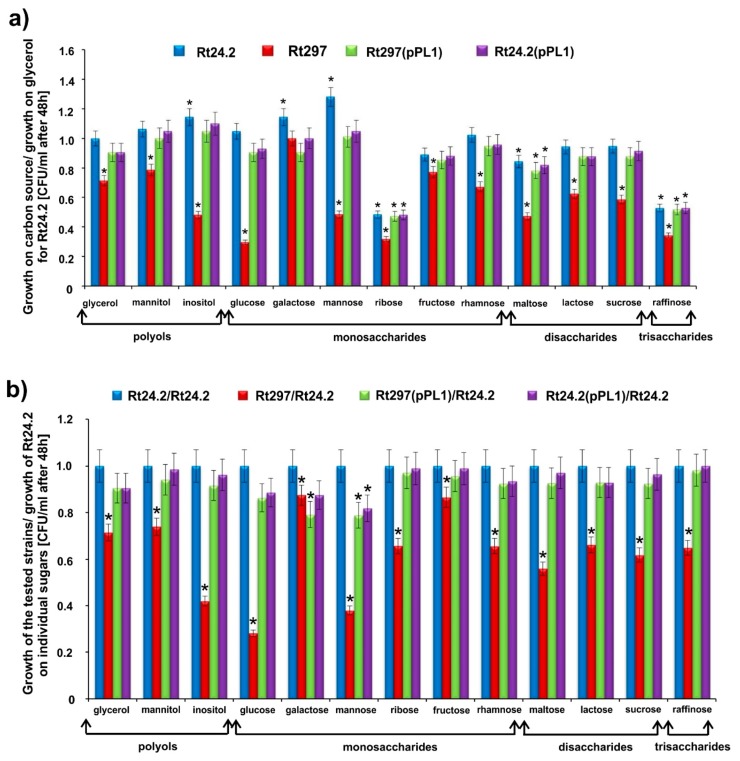
The utilization ability of different sugar compounds by the wild-type strain Rt24.2 and its derivatives, determined after 48 h of growth as culture CFU/mL. (**a**) Data are presented as a ratio of culture CFU/mL value of each individual strain tested in the presence of a particular carbon source per culture CFU/mL value for Rt24.2 grown in the presence of glycerol, which was chosen as a control Significant differences between the Rt24.2 culture with glycerol in relation to cultures with other carbon sources, as well as to cultures of other strains, are marked with * (*p*< 0.05, two-way ANOVA). (**b**) Data are presented as a ratio of culture CFU/mL value of the tested strains per culture CFU/mL value of Rt24.2 for particular sugars. Statistically significant differences between Rt24.2 and its derivatives were grown in the presence of individual sugars are marked with * (*p*< 0.05, one-way ANOVA).

Since the RNA-Seq data suggested that the functioning of some sugar transport systems might be impaired in *pssZ* mutant cells, the growth kinetics of the Rt297, Rt24.2, Rt297(pPL1) and Rt24.2(pPL1) strains was determined in the presence of various compounds (1%, *w*/*v*) selected as representatives of different sugar groups (Figure 5).

In general, the efficiency of utilization of the tested compounds, determined as CFU mL^−1^ values of 48-h cultures, was similar for the Rt24.2, Rt297(pPL1), and Rt24.2(pPL1) strains (no statistically significant differences between these strains were found with the only exceptions of galactose and mannose). Moreover, a great majority of the tested compounds (with the exception of ribose and raffinose) were effectively utilized by Rt24.2 and its derivatives Rt297(pPL1) and Rt24.2(pPL1) (Figure 5a,b). In contrast, the mutant Rt297 showed a significantly slower growth in the presence of the majority of the tested sugars (from 1.35-fold to 2.63-fold, depending on the compound tested) in comparison to the other strains. The exceptions were galactose, and fructose, for which the growth rate of the *pssZ* mutant was similar to that observed for the wild type. Furthermore, this mutant grew less effectively in the presence of inositol, glucose, mannose, ribose, lactose, sucrose, and raffinose, when compared to its growth in the presence of glycerol, which was chosen as a control carbon source (Figure 5a). Among the tested compounds, galactose, fructose, and mannitol were found to be more preferable sugars for the mutant’s growth. These data confirm that the *pssZ* mutation influences the effectiveness of the utilization of some sugars, probably by affecting the functioning of their transport systems. Transcriptomic analysis of Rt24.2 vs Rt297 strains showed that genes *Rt624_7* encoding a GDP-mannose-dependent-alpha-mannosyltransferase (log_2_ fold change 24.2/297 2.69), *Rt784_76* encoding a PTS mannose transporter (1.91), *Rt679_6* encoding a UDP-glucose 4-epimerase (12.70), and *Rt623_91* encoding an UDP-phosphate glucose phosphotransferase (1.80), were down-regulated in the *pssZ* mutant. Additionally, the expression of *Rt762_148* encoding a putative inositol utilization protein was decreased in this strain (log_2_ fold change 24.2/297 2.21). These phenotypic data confirm that the utilization efficiency of some sugar compounds by the *pssZ* mutant cells was reduced in comparison to that of the Rt297, Rt24.2, Rt297(pPL1), and Rt24.2(pPL1) strains, and this fact positively correlates with the RNA-Seq data.

#### 2.2.3. Synthesis of Different Polysaccharides

**Figure 6:**
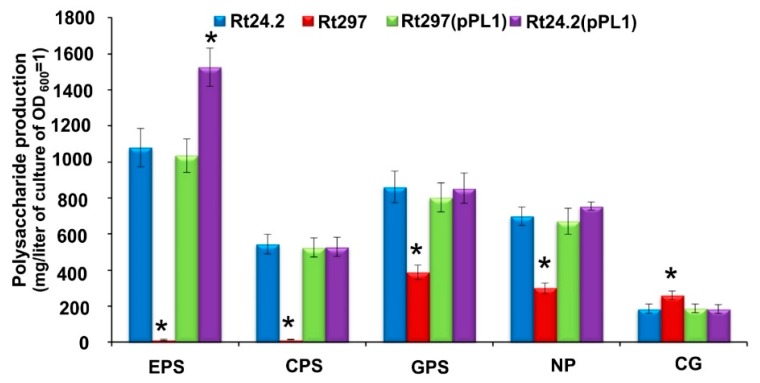
The amounts of different polysaccharides synthesized by the wild-type strain Rt24.2 and its derivatives Rt297, Rt297(pPL1), and Rt24.2(pPL1), determined as mg/litre of the culture of an optical density (OD_600_) =1. Significant differences between Rt24.2 and its derivatives in production of individual PSs are marked with * (*p*< 0.05, one-way ANOVA).

We also wanted to check whether the *pssZ* mutation affects the production of different rhizobial PSs present in *R. leguminosarum*. We previously established that the inactivation of *pssZ* totally inhibited EPS production (both LMW and HMW fractions), but did not affect the Rt297 LPS electrophoretic profile [59]. In this work, we determined the amounts of other rhizobial PSs produced by the wild-type strain Rt24.2 and its derivatives (Figure 6).

Our results show that Rt24.2, Rt297(pPL1), and Rt24.2(pPL1) synthesize similar amounts of the following PSs: CPS, GPS, NP, and CG. This fact indicates that the presence of additional *pssZ* copies in Rt24.2(pPL1) does not significantly affect the produced amounts of PSs (the only exception was EPS, for which a higher production in relation to the wild type was observed). In contrast, the *pssZ* mutant Rt297 does not produce EPS and CPS. Moreover, this strain synthesizes a lower amount of GPS (2.2-fold reduction) and NP (2.1-fold reduction), but a greater amount of CG (1.4-fold increase) than the wild type. Since CG plays an important role in osmoprotection, we decided to check the tolerance of the strains to high NaCl concentrations. Rt297 was found to be more resistant to high salt concentrations than the other tested strains. It was able to grow in the presence of 0.5% NaCl in contrast to the Rt24.2, Rt297(pPL1), and Rt24.2(pPL1) strains, which did not grow in these conditions (data not shown). In general, these findings positively correlate with the data obtained from our RNA-Seq analysis (Figure 2, Table S1), which indicate that the expression of several genes involved in EPS/CPS synthesis was strongly repressed in the *pssZ* mutant (e.g., a great majority of the genes located in the Pss-I region: *pssV, pssW, pssS, pssRMLKJ, pssI, pssF,* and *pssCDE*). Furthermore, the *Rt782_16*(*ndvA)* gene (involved in CG production) appeared as up-regulated in both the RNA-Seq (log_2_ fold change 24.2/297 −1.98) and β-galactosidase activity analyses in the *pssZ* background, whereas the *Rt627_76(gelA*) gene (involved in GPS synthesis) was down-regulated in this strain (Figure 2 and Figure 3). Similarly, the *Rt623_91* gene encoding an UDP-phosphate glucose phosphotransferase, which is an orthologue of *gmsA* from *R. leguminosarum* bv. *viciae* strain 3841 that is involved in NP synthesis, was down-regulated in the *pssZ* mutant (log_2_ fold change 24.2/297 1.80).

In conclusion, our results show that PssZ is involved in the regulation of mainly EPS and CPS synthesis in *R. leguminosarum*, but also affects the production of GPS and NP, and slightly that of CG.

## 3. Discussion

In our previous study, we characterized the *pssZ* gene of *R. leguminosarum* bv. *trifolii* located in the Pss-I region and proved that it is required for EPS synthesis [59]. This gene is an individual open reading frame, which does not form a part of any operon. In the *pssZ* upstream region, sequence motifs with a high identity to −35 and −10 hexamers recognized by *E. coli* δ^70^ RNA polymerase were found. Using *pssZ-gusA* genomic fusion and β-glucuronidase assay, the presence of a promoter in this region was confirmed [59]. However, the regulation of the *pssZ* expression has not been determined so far. The *pssZ* gene encodes a protein that shares significant similarity with serine/threonine protein phosphatases (STPs) from various bacterial species. To date, no proteins with such an enzymatic activity have been characterized in rhizobia. Several recent studies show that signalling systems composed of Ser/Thr kinases (STKs) and Ser/Thr phosphatases (STPs) play an important role in the bacterial regulatory network [60,72,73]. Although these systems do not have dedicated transcription factors, they affect gene expression by phosphorylation/dephosphorylation of different regulatory proteins. Phosphoproteomic analyses in various Gram-positive and Gram-negative bacteria as well as in *Archaea* identified numerous (~100) proteins phosphorylated on Ser or Thr residues, indicating that the regulation of gene expression based on the STK and STP enzymes is common in prokaryotic microorganisms [62,74].

We showed earlier that inactivation of *pssZ* causes pleiotropic effects in rhizobial cells, including the inhibition of EPS synthesis, reduced growth kinetics and motility, and failure in clover root infection [59]. These findings suggested that *pssZ* might play a global regulatory role in the functioning of *R. leguminosarum* bv. *trifolii* cells, not only in symbiosis but also in free-living conditions. This fact prompted us to carry out a comparative transcriptomic analysis of the wild-type strain Rt24.2 and the *pssZ* mutant Rt297 in order to identify genes differentially expressed (DEGs) in these two genetic backgrounds. Using the RNA-Seq approach, a large group of genes (996) was identified as belonging to the PssZ regulon. Among these genes, the most numerous were those related to transcription, signal transduction, carbohydrate transport and metabolism, and cell wall/membrane/envelope biogenesis (COGs K, T, G, and M) (Figure 1). Among these DEGs, many genes encoding transcriptional factors belonging to different regulatory families and those engaged in cellular signalling were either down- or up-regulated in the *pssZ* mutant (Figure 2, Table S1). Furthermore, the expression of a high number of genes involved in EPS synthesis was found to be affected by the *pssZ* mutation, and most of them were strongly down-regulated or even repressed in the mutant. These include a great majority of the genes located in the Pss-I region, which encode glycosyl transferases involved in the EPS subunit assembly (e.g., *pssDE, pssC, pssS, pssF, pssG, pssH,* and *pssI*) and proteins adding non-sugar modifications to EPS (*pssM, pssK, pssR*) (Figure 2). However, the expression of other genes from this region *(pssT, pssN, pssO,* and *pssP)*, which are involved in EPS polymerization and export, were not affected by the *pssZ* mutation. Interestingly, *pssA*, which is located ~90 kb from the EPS-I region and codes for the glycosyl transferase that initiates EPS synthesis, was moderately up-regulated in the *pssZ* mutant. To our knowledge, this is the first report that shows the participation of the STP-type protein in the regulation of EPS synthesis in bacteria. Our data also indicate that the lack of EPS synthesis observed in the Rt297 strain was most probably caused by either the repression or lack of activation of the genes involved in the EPS subunit assembly, in which some transcriptional factors might be engaged. In fact, the PssZ regulon includes numerous transcriptional regulators belonging to different families, and possibly one or some of these proteins could be actually involved in the regulation of EPS-related genes. Our finding provides new insights into the regulation of EPS synthesis in *R. leguminosarum*, in which other proteins besides RosR might be also engaged in positive regulation of this process (since, surprisingly, *rosR* was not repressed but slightly up-regulated in the *pssZ* mutant). Our results suggest that some of the regulatory protein/proteins involved in EPS production might be substrates for the PssZ phosphatase. Besides three putative STKs (Rt622_94, Rt626_101(PrkA), and Rt645_26(HprK)), transcriptional regulators from different families (e.g., Rt688_7 (Cro/Cl family), Rt678_1 and Rt713_1 (LysR), Rt787_17 (AraC), Rt764_66 (DeoR), Rt619_151 (ROK), and Rt782_65 (GntR)) might be also possible targets for PssZ. The transcriptome profile of the *pssZ* mutant also revealed changes in the expression of several other genes related to cell wall/membrane/envelope biogenesis (COG M) (Figure 2). These results explain our earlier observations of changed colony morphology (rough colonies), topography, and surface properties of *pssZ* mutant cells. As established in AFM analyses, these cells were larger and their surface was smoother, less hydrophobic and more inflexible than the wild-type cells [59]. Furthermore, Rt24.2 and its derivatives Rt297(pPL1) and Rt24.2(pPL1) produced large amounts of various rhizobial PSs under the tested growth conditions, whereas the *pssZ* mutant did not synthesize EPS and CPS, and produced diminished amounts of GPS and NP but slightly increased CG yield (Figure 6). The fact that the inactivation of a single gene could affect the production of different rhizobial polysaccharides has been previously reported. This is the case, for example, of the inactivation of *pssA* in *R. leguminosarum* strain 3841, which avoided the production of both EPS and CPS [23]. Among the DEGs detected in our study, other genes potentially engaged in the synthesis and export of different PSs, *ndvA* involved in CG synthesis, and *gmsA* involved in NP synthesis were found (Figure 2 and Figure 3, Table S1). Similarly, an *exo5* mutant of *R. leguminosarum* RBL5523, defective in a UDP-glucose dehydrogenase, was unable to produce both EPS and CPS and lacked galacturonic acid residues in its LPS [75]. In *S. fredii* HH103, a lack of the UDP-glucose dehydrogenase encoded by *rkpK* also resulted in the absence of EPS production and in alterations in LPS [76]. As reported by us earlier, the *pssZ* mutation affected EPS production in *R. leguminosarum* bv. *trifolii*, resulting in total inhibition of the synthesis of both LMW and HMW fractions, but it did not affect the LPS profile [59]. The lack of EPS caused strong symbiotic defects in the *pssZ* mutant on clover plants similarly to other EPS-deficient *R. leguminosarum* and *S. meliloti* strains (i.e., dramatically reduced competitiveness and inability to infect host plant roots) [23,31,32,33,75]. EPS, which forms the most external layer of bacterial cells, plays an important protective role against several stress factors occurring in soil. Moreover, CGs located in the periplasmic space are important for rhizobial osmoprotection [77]. In this study, we show that the *pssZ* mutant exhibits increased CG production and higher tolerance to NaCl concentrations than the wild-type strain. Similarly, previous studies performed in *S. fredii* HH103 demonstrated that the inactivation of *mucR1,* encoding a positive regulator of EPS synthesis, resulted not only in a lack of EPS biosynthesis but also in an increase in extracellular CG production [78], and that the inactivation of the CG synthase *ndvB* provoked increased EPS production [79]. All these results suggest that some rhizobia have developed mechanisms that are able to compensate for the lack of either EPS or CG with enhanced production of the other PS. But this is not always the case since a mutation of the sensor kinase *chvG* in *R. leguminosarum* bv. *viciae* VF39 caused, similarly to the *pssZ* mutant of Rt24.2, pleiotropic phenotypes, negatively impacting cellular metabolism, membrane stability and symbiosis with its host plants [80]. The VF39 *chvG* mutant produced nearly 2-fold fewer EPS and neutral PSs, and induced nodules unable to fix nitrogen. This effect was caused by the down-regulation of the *pssA* and *ndvB* genes in the mutant.

The transcriptome profiling of the *pssZ* mutant also revealed that the expression of a large group of genes (14), encoding putative diguanylate cyclases involved in the synthesis of cyclic di-GMP (c-di-GMP), was down-regulated in this strain (Table S1). These data suggest that this signal molecule might be engaged in the regulation of EPS synthesis in *R. leguminosarum.* As reported previously, the synthesis of various PSs in several bacterial species is regulated by this second messenger. Recent data show that high intracellular c-di-GMP concentrations favour EPS production, the formation of fimbriae and pili, and play a role in quorum-sensing, cell cycle control, and virulence regulation [81,82,83]. For example, c-di-GMP stimulates cellulose synthesis in *Pseudomonas syringae* pv. tomato DC3000 [84] and *Gluconacetobacter xylinus* [70,71], as well as the synthesis of PSs in *Vibrio cholerae* [68]. It was established that c-di-GMP is recognized and bound by some transcription regulators in these bacteria. For example, c-di-GMP is required for the dimerization of an activator VpsT in *V. cholera* [68]. Moreover, it was found that the overproduction of a diguanylate cyclase in *R. etli* and *R. leguminosarum* enhanced EPS production, biofilm formation, and adhesion to plant roots [85]. In *S. meliloti,* the role of c-di-GMP-related genes in affecting the growth rate, motility, EPS production, and nodule occupancy was confirmed [66,86]. As recently shown for this bacterium, increased c-di-GMP levels induced the production of a novel mixed-linkage β-glucan, by binding this molecule to a membrane-bound glycosyl transferase BgsA [87,88]. These data suggest that c-di-GMP levels might be also engaged in an indirect manner in the regulation of EPS production in *R. leguminosarum* cells.

In this study, we also examined the growth of the *pssZ* mutant and the wild-type strains at a wide range of temperatures (16–32 °C). However, no significant differences in tolerance to low (16 °C), and only slightly higher tolerance to high (32 °C) temperatures for the mutant in relation to the remaining tested strains were found. It is known that the most frequently involved chaperones of the thermal shock response are those from the DnaK-DnaJ-GrpE and GroES-GroEL systems [89,90]. We found that the mutation in the *pssZ* affected expression of several genes encoding proteins of such putative functions in *R. leguminosarum,* and a majority of them were up-regulated in the mutant background (Figure 2, Table S1). This fact suggests that the presence of these protective proteins at higher levels might increase the tolerance of Rt297 to elevated temperature (32 °C) in relation to the other studied strains (that was noticed after 72-h incubation).

We also showed in this study that the *pssZ* mutation affected the expression of a large group of genes related to the transport and metabolism of various nutrients (COGs C, E, F, G, H, I, P, Q), especially those associated with sugar uptake (COG G) (Figure 2 and Figure 3, Table S1). The results from the RNA-Seq analysis were in coherence with the growth experiments using several representative compounds as a carbon and energy source (Figure 5). Our data suggest that PssZ plays an important role in the regulation of many metabolic genes in *R. leguminosarum*. We found that the *pssZ* mutation affected several rhizobial genes involved in transport and utilization of carbohydrates. For example, this mutation resulted in reduced efficiency of utilization of monosaccharides such as glucose or mannose, as well as all tested disaccharides (lactose, maltose and sucrose) and inositol (Figure 5). The proper functioning of metabolic pathways is extremely important for all microorganisms. Several authors reported that an ability to utilize a high number of carbon and energy sources plays an important role in the adaptation of rhizobia to both soil conditions and their competitiveness in host plant infection [91,92,93]. The presence of a large number of genes related to carbon sources uptake in rhizobial genomes provides metabolic plasticity and enables rhizobia to survive in the complex soil environment, as well as inside plants [94,95]. For example, catabolism of homoserine, an important component of pea root exudate by *R. leguminosarum* bv. *viciae* 3841, was found to be associated with its competitiveness for nodulation of this host plant [96]. Rhizobia are characterized by extremely large genomes (up to 9 Mbp), which besides the chromosome contain several large plasmids, that ensures them high metabolic plasticity [97]. As reported recently, rhizobial strains utilizing a wider range of substrates (including sugar substrates) are more competitive than others and, as a consequence, are more successful in symbiosis [98]. The diverse metabolic capacities of rhizobial strains are important for the adaptation to soil and survival in the rhizospheres of host plants. Legume root exudates contain a high number of compounds, including sugars, amino acids, amines, aliphatic and aromatic acids, and others [99,100]. Our results suggest that PssZ might play an important role in the rhizobial adaptation to both soil conditions and symbiosis with host plants.

## 4. Materials and Methods

### 4.1. Bacterial Strains, Plasmids, and Culture Conditions

Bacterial strains, plasmids, and oligonucleotide primers used in this work are listed in Table 1.

*R. leguminosarum* strains were cultured in a 79CA medium with 1% glycerol (*w*/*v*) as a carbon source at 28 °C on a rotary shaker (200 rpm) [105], whereas *E. coli* strains were grown in Luria-Bertani (LB) medium at 37 °C [106]. When required, antibiotics were used at the following final concentrations: spectinomycin, 40 μg mL^−1^; rifampicin, 40 μg mL^−1^; nalidixic acid, 40 μg mL^−1^; tetracycline, 10 μg mL^−1^; kanamycin, 40 μg mL^−1^ (for rhizobial strains, 40 μg mL^−1^ for agar plates and 20 μg mL^−1^ for cultures were used). To determine the growth kinetics of Rt24.2, the Rt297, Rt297(pPL1), and Rt24.2(pPL1) strains at different temperatures, bacterial cultures in 79CA of an initial optical density (OD_600_) = 0.1 were prepared. In the case of Rt297(pPL1) and Rt24.2(pPL1) strains, kanamycin was added. The cultures were incubated at 16, 20, 24, 28, and 32 °C for 72 h with shaking at 200 rpm. After each 24 h, culture OD_600_ was measured, and then 100-μL aliquots were taken and placed in serial dilutions onto 79CA agar plates. The bacterial colonies (colony-forming units, CFU) appearing after 3-day incubation at 28 °C were counted. The experiment was repeated twice with three biological replicates for each strain and condition tested.

Growth kinetics in the presence of different sugars was studied using bacterial cultures in 79CA of the initial OD_600_ = 0.1, which were incubated for 48 h at 28 °C. After 24 and 48 h, culture OD_600_ was measured and then 100-μL aliquots were placed in serial dilutions on 79CA agar plates, and after 72-h incubation, CFU was counted. The experiment was carried out twice with three biological replicates for each strain and condition tested.

### 4.2. Isolation of Total RNA and Synthesis of cDNA Libraries

The isolation of total RNA from *R. leguminosarum* strains was performed according to a method described earlier [58]. Briefly, 25-mL cultures of Rt24.2 and Rt297 grown for 24 h in 79CA were centrifuged (12,000× *g*, 15 min) and bacterial pellets obtained were suspended in 15 mL Trizol, shaken vigorously, and incubated for 5 min at room temperature. Then, 3 mL of chloroform was added to each mixture, shaken vigorously (15 s), incubated at room temperature (8 min), and subsequently centrifuged (12,000× *g*, 15 min, 4 °C). RNA present in a water phase was precipitated using isopropanol (2:1, *v*/*v*) by incubation at room temperature (15 min) and centrifugation (12,000× *g,* 15 min, 4 °C). RNA pellets were washed twice with 1 mL 75% ethanol, dried, and dissolved in deionized RNase- and DNase-free water (10 min, 55 °C). The RNA concentration and quality in samples were determined spectrophotometrically using NanoDrop 2000 (Thermo Fisher Scientific, Waltham, MA, USA). DNA traces from RNA were removed using a TURBO DNA-free Kit (Thermo Fisher Scientific) according to a manufacturer’s instruction. Possible contamination of RNA by DNA was checked using PCR and primers complementary to *R. leguminosarum pssY* (pssY5f and pssY5r) and *pssA* (pssAG1f and pssA2r) genes (Table 1). For PCR, a REDTaq Ready PCR Reaction Mix (Sigma-Aldrich, St. Louis, MO, USA) was used. rRNA from total RNA was removed using a Ribo-Zero Magnetic Kit for Gram-Negative bacteria (Epicentre, Illumina, San Diego, CA, USA). rRNA-depleted mRNA was precipitated using ice-cold ethanol (3:1, *v*/*v*). For this purpose, the samples were incubated for 60 min at −20 °C, and next centrifuged (12,000× *g,* 30 min, 4 °C). Pellets were washed twice using ice-cold 75% ethanol, centrifuged (12,000× *g,* 5 min), and dissolved in RNase- and DNase-free water. The mRNA obtained was quantified spectrophotometrically and its integrity was assessed using an RNA 6000 Pico Kit and Agilent Bioanalyzer 2100 (Agilent Technologies, Santa Clara, CA, USA). Three independent mRNA isolations (i.e., biological repeats) were done for each strain. Transcriptome libraries were prepared using a NEBNext Ultra Directional RNA Library Prep Kit for Illumina (New England, BioLabs, Hitchin, UK) following the manufacturer’s protocol.

### 4.3. RNA-Seq Data Analysis

For transcriptomic analyses, cDNA libraries obtained for Rt24.2 and Rt297 were sequenced using a MiSeq System with SBS technology (Illumina), with three independent biological replicates performed for each strain. Preliminary preparation of reads for analysis, including the elimination of adapters and low-quality reads, were done using Trimmotatic software (operating mode for paired-end) (Illumina, Phred+33) [107]. The remaining reads for both Rt24.2 and Rt297 strains were then mapped using Bowtie2 (within the Tophat package) [108] and Rt24.2 genome as a reference genome [58]. The median read number per CDS was above 1,000 (log value >3). Next, numbers of reads mapped to individual genes were calculated using HTseq programme [109]. Final results were analyzed in the R environment using the DEseq2 package [110,111]. On average, 14,885,607 reads for the wild-type strain (K1 = 15,927,160; K2 = 14,848,310; K3 = 13,881,353; SD = 835,613.51) and 14,667,946 reads for the *pssZ* mutant (J1 = 14,835,882; J2 = 16,189,714; J3 = 12,978,242; SD = 1,316,444.701) were obtained, indicating that similar amounts of data were mapped for each strain studied.

For the identification of genes of statistically significant differences in expression between the Rt24.2 and Rt297 strains, the significance threshold value was set to 0.05 (using the Benjamini-Hochberg False Discovery Rate (FDR) correction; Wald test) [108,112]. CDS with FDR-corrected *p* values for different expressions between the tested strains lower than 0.05 were considered significant. A list of genes differentially expressed with fold changes in the wild type versus the *pssZ* mutant was obtained, and the normalized expression was presented as the number of reads for an individual gene normalized per a total library size for a particular sample. To classify genes differentially expressed into functional categories, Clusters of Orthologous Groups (COG) database was used [113].

### 4.4. Analysis of Transcriptional Fusions

Transcriptional fusion plasmids containing promoter regions of rhizobial genes cloned upstream of reporter *lacZ* or *gusA* genes (Table 1) were transferred from *E. coli* S17-1 to Rt24.2 and Rt297 strains by bi-parental conjugation. For this purpose, 24-h cultures of *E. coli* S17-1 derivatives carrying fusion plasmids (donor strains) and Rt24.2 and Rt297 (recipient strains) were mixed in a 1:10 ratio (*v*/*v*) and centrifuged (6000× *g,* 10 min). Next, bacterial pellets were washed twice in 1 mL of sterilized water and the obtained mixtures were centrifuged. Finally, the pellets were suspended in 0.2 mL of water, placed on 79CA agar plates, and incubated for 48 h at 28 °C. Then, the bacteria were collected from the plates to 1 mL of sterilized water and spread in 0.1-mL aliquots on 79CA agar plates supplemented with rifampicin and tetracycline. Transconjugants obtained after a 7-day incubation were used for the determination of the transcriptional activity of the tested promoters. β-galactosidase/β-glucuronidase activity assay was carried out according to Miller’s protocol [114] using 2-nitrophenyl-β-D-galactopyranoside (ONPG) or p-nitrophenyl-β-D-glucuronide (NPG) as a substrate for β-galactosidase and β-glucuronidase, respectively (Sigma-Aldrich). For this assay, 24-h cultures of Rt24.2 and Rt297 derivatives containing transcriptional fusions were used. Rt24.2 and Rt297 strains containing empty pMP220 and pFUS1P vectors were used as a control. To avoid the influence of EPS on culture optical density, the cultures were centrifuged before being used for the assay (6000× *g,* 10 min). Bacterial pellets were suspended in a buffer Z [114] and the OD_600_ of suspensions were measured. Next, 20 μL chloroform and 20 μL 0.1% SDS (*w*/*v*) were added to 1 mL of bacterial suspensions (*v*). Samples were shaken for bacterial lysis and evaporation of chloroform (20 min). A total of 200 μL of ONPG or NPG (4 g L^−1^ in buffer Z) was added and the samples were incubated for 5 min (*t*) at 37 °C. The reaction was stopped by adding 500 μL of 1 M Na_2_CO_3_. Next, the samples were centrifuged (10,000× *g,* 7 min) and their 300-μL aliquots were added to titration plate wells, and the OD_420_ was measured (Asys UVM 340, Biochrom, Cambridge, UK). The assay was done in triplicate for each strain tested with three biological repetitions. The activity of β-galactosidase/β-glucuronidase was calculated according to the following formula and presented as Miller units:

β-galactosidase/β-glucuronidase activity (Miller units) = (1000×𝑂𝐷_420_)/(*t* ×𝑣 × 𝑂𝐷_600_)

### 4.5. Isolation of Surface Polysaccharides

#### 4.5.1. EPS

For EPS isolation, 5-mL cultures of the Rt24.2, Rt297, Rt297(pPL1), and Rt24.2(pPL1) strains were grown in 79CA for 72 h. After this time, OD_600_ of each culture was measured and its 1.5-mL aliquots were centrifuged (12,000× *g,* 15 min). EPS was precipitated from the culture supernatant at 4 °C overnight using cold 95% ethanol (a 1:4 ratio for HMW and a 1:10 ratio (*v*/*v*) for LMW EPS, respectively). Next, the samples were centrifuged (12,000× *g,* 20 min), and the EPS obtained was dried, suspended in deionized mili-Q water, and analyzed using an indole-sulphuric acid method [115]. The total sugar content was calculated as glucose equivalents. The experiment was carried out twice with three replicates for each strain.

#### 4.5.2. Gel-Forming Polysaccharide

The bacterial pellet obtained from 100 mL of a 5-day culture was suspended in 20 mL of deionized water. Next, 20 mL of 2N NaOH was added to the bacterial suspension and mixed for 1.5 h at room temperature. Bacterial cells were removed by centrifugation (8000× *g,* 30 min, 4 °C) and the supernatant was acidified by addition of acetic acid. Precipitated GPS was collected by centrifugation, dried, dissolved in deionized mili-Q water, and analyzed according to Reference [115]. The experiment was done twice with three replicates for each strain.

#### 4.5.3. Capsular Polysaccharide

This PS was isolated from the bacterial pellet obtained from 100 mL of 5-day cultures. The pellet was suspended in 20 mL of 1N NaOH and the mixture was agitated for 1.5 h at room temperature. CPS was precipitated by the addition of cold 95% ethanol (1:1, *v*/*v*), and collected by centrifugation (8000× *g,* 30 min, 4 °C). Next, CPS was dried, dissolved in deionized mili-Q water, and analyzed according to [115]. The experiment was performed twice with three replicates for each strain tested.

#### 4.5.4. Cyclic β-Glucans

For isolation of cyclic β-glucans, supernatants remaining from CPS isolation (which contained 50% ethanol) was used. The glucose concentration in the supernatants was determined according to Reference [115]. The experiment was performed twice with three replicates for each strain tested.

#### 4.5.5. Glucomannan

The isolation of NP was performed according to a method described in Reference [21]. Briefly, the bacterial pellet obtained from 1 L of a 5-day culture (79CA medium) was extracted by the hot phenol-water method with several modifications [116]. The obtained water phase was then dialyzed against water using a dialysis tube (12-14 kDa) and lyophilized. The material was then suspended in a binding buffer (100 mM NH_4_HCO_3_, pH 8.0 and 0.9% NaCl) and applied to a polymyxin B column in a ratio of 30 mL of material per 10 mL of bed (incubation overnight to bind LPS). Glucomannan (NP) was then eluted from the column using the binding buffer (at a rate of 5 mL per h), dialyzed and lyophilized. The experiment was performed twice with two replicates for each strain. The glucose concentration in the supernatants was determined according to Reference [115].

#### 4.5.6. Determination of PS Amounts Synthesized by Rhizobial Strains

The amounts of produced PSs were determined using an indole-sulphuric acid method [115]. For this assay, 20-μL aliquots of PS solutions were added to 500 μL of 75% H_2_SO_4_ and 20 μL of 1% indole dissolved in 95% ethanol (*w*/*v*). Samples were incubated for 15 min at 100°C, and 100-μL aliquots were added to titration plate wells, and their optical density (OD_470_) was measured. The assay was performed in triplicate for each sample analyzed. The results of the experiment were calculated using a curve done for glucose, whose function factor was determined on 0.0023.

### 4.6. Statistical Analysis

Statistical data analyses were performed using one-way analysis of variance (ANOVA) (Statistica, ver.12, StatSoft, Cracov, Poland), and significant differences between the analyzed samples were established at *p* < 0.05.

## 5. Conclusions

*Rhizobium leguminosarum* bv. *trifolii* is a soil bacterium able to establish nitrogen-fixing symbiosis with clover plants (*Trifolium* spp.). Comparative transcriptomic analyses of the *R. leguminosarum* bv. *trifolii* wild-type strain Rt24.2 and its derivative Rt297, carrying a mutation in the *pssZ* gene, allowed us to identify a large group of genes differentially expressed in these two genetic backgrounds. Our data confirmed the significance of PssZ in several cellular processes, including the synthesis of cell-surface polysaccharides, transcription regulation, cell signalling, and bacterial metabolism. This fact indicated that this putative serine-threonine phosphatase plays an important role in regulatory networks of *R. leguminosarum,* that are important for both symbiotic and free-living conditions. To our knowledge, this is the first study reporting the involvement of an STP protein in the expression of genes related to EPS production in a rhizobial strain.

## Figures and Tables

**Table 1:** The strains, plasmids and oligonucleotide primers used in this study.

Strains, Plasmids, and Primers	Characteristics	Source or Reference
***Strains***		
Rt24.2	wild-type strain *Rhizobium leguminosarum* bv. *trifolii,* clover microsymbiont, Rif^r^, Nx^r^	[101]
Rt297	Rt24.2 *pssZ*::mTn*5*SS*gusA40*, Sp^r^	[59]
Rt297(pPL1)	Rt297 carrying *pssZ* on pBBR1MCS-2 vector, Km^r^	[59]
Rt24.2(pPL1)	Rt24.2 carrying *pssZ* on pBBR1MCS-2 vector, Km^r^	[59]
Rt24.2(pMP220)	Rt24.2 carrying pMP220 vector, Rif^r^, Nx^r^_,_Tc^r^	This work
Rt297(pMP220)	Rt297 carrying pMP220 vector, Rif^r^, Nx^r^_,_Tc^r^	This work
***Plasmids***		
pMP220	IncP, *mob*, promoterless *lacZ*, Tc^r^	[102]
pFUS1P	pFUS1 with *par* cassette, promoterless *gusA*, Tc^r^	[103]
pPL1	pBBR1MCS-2 carrying 1.8-kb *Sal*I-*Xba*I fragment with the *pssZ* gene, Km^r^	[59]
pPSS4	pMP220 carrying 0.6-kb *EcoR*I-*Pst*I fragment of the *pssB* promoter region	[58]
pNDV5	pMP220 carrying 0.3-kb *EcoR*I-*Pst*I fragment of the *ndvA* promoter region	[58]
pCEL9	pMP220 carrying 0.72-kb *EcoR*I-*Pst*I fragment of the *celA* promoter region	[58]
pGEL10	pMP220 carrying 0.8-kb*Bgl*II-*Xba*I fragment of the *gelA* promoter region	[58]
pRAP11	pMP220 carrying 0.9-kb *Bgl*II-*Xba*I fragment of the *rapA1* promoter region	[58]
pPRS12	pMP220 carrying 0.85-kb *EcoR*I-*Xba*I fragment of the *prsD* promoter region	[58]
pF65	pMP220 carrying 0.65-kb *Bgl*II-*Pst*I fragment of the *pssF* promoter region	[45]
pW74	pMP220 carrying 0.74-kb *EcoR*I-*Pst*I fragment of the *pssW* promoter region	[45]
pK48	pMP220 carrying 0.48-kb *EcoR*I-*Pst*I fragment of the *pssK* promoter region	[45]
pV90	pMP220 carrying 0.9-kb *KpnI*-*Xba*I fragment of the *pssV* promoter region	[45]
pC55	pMP220 carrying 0.55-kb *EcoR*I-*Sph*I fragment of the *pssC* promoter region	[45]
pO66	pMP220 carrying 0.65-kb *Bgl*II-*Pst*I fragment of the *pssO* promoter region	[45]
pN76	pMP220 carrying 0.75-kb *Bgl*II-*Pst*I fragment of the *pssN* promoter region	[45]
pT80	pMP220 carrying 0.8-kb *Bgl*II-*Pst*I fragment of the *pssT* promoter region	[45]
pP85	pMP220 carrying 0.85-kb *EcoR*I-*Xba*I fragment of the *pssP* promoter region	[45]
pI90	pMP220 carrying 0.9-kb *EcoRI-SphI* fragment of the *pssI* promoter region	[45]
pPA2	pMP220 carrying 0.9-kb *EcoR*I-*Xba*I fragment of the *pssA* promoter region	[32]
pEP1	pMP220 carrying 0.65-bp *EcoR*I-*Pst*I fragment of the *rosR* promoter region	[101]
pDGRP	pFUS1P carrying *mcpD-gusA* fusion	[103]
pCGR	pFUS1P carrying *mcpC-gusA* fusion	[103]
**Primers**	**Sequence (5′→3′)**	
pssAG1f	CGCACATGCGAAAGATTTGCTGCG	[104]
pssA2r	CCAGATCGAGGAATTCCCGACGTA	[104]
pssY5f	GTCGTCGATGACGATGCGGCTGTT	[104]
pssY5r	GAAACTATGTGCTTCCCATGTCATCG	[104]

Rif^r^- rifampicin, Nx^r^- nalidixic acid, Sp^r^- spectinomycin, Tc^r^- tetracycline, Km^r^– kanamycin.

## Supplementary Materials

Supplementary materials can be found at https://www.mdpi.com/1422-0067/20/12/2905/s1.

## Abbreviations

EPS	exopolysaccharide
LPS	lipopolysaccharide
PS	polysaccharide
LMW	low-molecular-weight
HMW	High-molecular-weight
CPS	capsular polysaccharide
NP	neutral polysaccharide
GPS	gel-forming polysaccharide
CG	cyclic β-glucan
IT	infection thread
STP	serine/threonine protein phosphatases
STK	Hanks-type serine/threonine kinase
DEG	differentially expressed gene
COG	cluster of orthologous group
