# Supplementary material for: Transcriptomic Studies Reveal that the Rhizobium leguminosarum Serine/Threonine Protein Phosphatase PssZ has a Role in the Synthesis of Cell-Surface Components, Nutrient Utilization, and Other Cellular Processes
Source: Int J Mol Sci. 2019 Jun 14;20(12):2905. doi: 10.3390/ijms20122905 (PMC6628131; doi:10.3390/ijms20122905)
Supplement: Supplementary file 1 [file ijms-20-02905-s001.zip › Supplementary Table S2.pdf]

Supplementary material
Table S2

A list of differentially expressed genes (DEGs) of the highest values (log_2 fold-change Rt24.2/Rt297 ≥5 or ≤-5)

Gene	Function	Gene expression (log_2 fold change $_{Rt24.2/Rt297}$)
	**Carbohydrate transport and metabolism (COG G)**	
Rt651_4	L-xylulose kinase	-9.02
Rt651_5	aldolase	-7.81
Rt651_6	glycerol-3-phosphate dehydrogenase	-7.23
Rt651_7	aldo/keto reductase	-9.58
Rt651_9	ABC transporter permease	-7.36
Rt651_10	D-ribose transporter ATP-binding protein	-8.36
Rt651_11	ABC transporter	-10.50
Rt651_20	major facilitator transporter	-10.70
Rt651_22	pyridoxamine 5-prime-phosphate	-10.36
Rt651_41	4-alpha-glucanotransferase	-9.39
Rt659_5	ribokinase	-10.12
Rt659_7	ribose ABC transporter permease	-8.49
Rt659_8	ribose ABC transporter	-8.19
Rt659_9	RbsB	-9.19
Rt659_11	ribokinase	-9.33
Rt659_12	fucose binding protein	-8.85
Rt659_13	sugar ABC transporter permease	-9.97
Rt659_14	sugar ABC transporter permease	-10.16
Rt659_15	sugar ABC transporter substrate-binding protein	-11.12
Rt659_16	sugar ABC transporter ATP-binding protein	-8.79
Rt659_17	fucose binding protein	-12.13
Rt679_5	glycosyl transferase	12.27
	**Amino acid transport and metabolism (E)**	
Rt651_21	divalent metal transporter permease	-12.03
Rt651_29	D-amino acid oxidase	-9.26
Rt651_30	amino acid ABC transporter ATP-binding protein	-8.84
Rt651_31	amino acid ABC transporter substrate-binding protein	-9.11
Rt659_2	cytochrome C550	-7.37
Rt659_3	peptide ABC transporter ATP-binding protein	-8.04
Rt659_4	ABC transporter	-8.93
Rt659_20	glycine/betaine ABC transporter	-11.02
Rt659_21	glycine/betaine ABC transporter	-10.69
Rt659_22	glycine/betaine ABC transporter permease	-10.42
Rt659_24	ornithine cyclodeaminase	-12.13
Rt659_26	histidine ammonia-lyase	-10.72
Rt772_15	acyl-CoA thioesterase	10.98
Rt782_61	cytochrome C4	6.02
Rt782_62	glutamine ABC transporter ATP-binding protein	6.42
Rt782_63	ABC transporter permease	7.00
Rt782_64	ABC transporter substrate-binding protein	6.11
	**Lipid transport and metabolism (I)**	
Rt621_23	S-adenosylmethionine: diacylglycerol 3-amino-3-carboxypropyl transferase	5.09
Rt651_3	choline dehydrogenase	-9.64
Rt651_17	monooxygenase	-11.67
Rt651_24	phospholipase D	-10.64
Rt659_28	dehydratase	-10.52
Rt659_29	oxidoreductase	-9.79
Rt659_30	CoA transferase	-11.20

**Inorganic ion transport and metabolism (P)**		
Rt659_2	cytochrome C550	-7.37
Rt659_3	peptide ABC transporter ATP-binding protein	-8.04
Rt659_4	ABC transporter	-8.93
Rt659_23	phosphate ABC transporter substrate-binding protein	-9.98
**Secondary metabolites biosynthesis, transport, and catabolism (Q)**		
Rt634_68	phasin	-6.09
Rt651_14	6-chlorohydroxyquinol-1,2-dioxygenase	-7.24
**Energy production and conversion (C)**		
Rt651_15	maleylacetate reductase	-8.69
Rt651_18	4-hydroxyphenylacetate 3-monooxygenase	-7.51
**Coenzyme transport and metabolism (H)**		
Rt621_24	methyltransferase	5.08
Rt688_8	3-demethylubiquinone-9 3-methyltransferase	11.07
Rt759_1	3-phosphoglycerate dehydrogenase	5.96
**Translation, ribosomal structure and biogenesis (J)**		
Rt724_2	30S ribosomal protein S21	-8.19
**Transcription (K)**		
Rt651_2	cold-shock protein	-12.65
Rt651_8	Crp/Fnr family transcriptional regulator	-9.87
Rt651_32	LacI family transcriptional regulator	-11.03
Rt651_33	LuxR family transcriptional regulator	-12.65
Rt659_6	RpiR family transcriptional regulator	-10.71
Rt659_27	histidine utilization repressor	-10.65
Rt659_32	LysR family transcriptional regulator	-10.87
Rt688_7	Cro/Cl family transcriptional regulator	12.66
Rt713_1	LysR family transcriptional regulator	5.78
Rt770_14	TetR family transcriptional regulator	-5.13
**Cell wall/membrane/envelope biogenesis (M)**		
Rt659_10	xylanase	-9.37
Rt659_25	ABC transporter	-12.62
Rt679_1	glycosyl transferase family A	12.19
Rt679_3	glycosyl transferase	10.95
Rt679_4	glycosyl transferase	12.92
Rt679_6	UDP-glucose 4-epimerase	12.70
Rt772_1	exopolysaccharide biosynthesis protein	9,79
Rt772_2	membrane protein	9,57
Rt772_3	1,4-beta-xylanase	13.33
Rt772_4	glycosyl transferase	15.10
Rt772_5	acetyltransferase	11.96
Rt772_6	ketal pyruvate transferase	12,48
Rt772_7	exopolysaccharide biosynthesis protein	14.42
Rt772_8	exopolysaccharide biosynthesis protein	13.89
Rt772_9	galactosyl transferase	13,09
Rt772_10	glycosyl transferase	13.07
Rt772_11	glycosyl transferase	13.23
Rt772_12	glycosyl transferase family A	12.07
Rt772_13	glucuronosyl transferase	12.95
Rt772_14	glucuronosyl transferase	12.59
Rt772_15	hypotetical protein (Orf3)	10.980
Rt772_16	hemolysin secretion protein D	13.217
Rt772_17	type I secretion protein	14.073
Rt772_18	polysaccharidase	13.23
Rt772_19	autoaggregation protein	13,22
**Signal transduction mechanisms (T)**		
Rt651_28	chemotaxis protein	-9.59
Rt651_31	amino acid ABC transporter substrate-binding protein	-9.11

Rt688_6	peptide antibiotic resistance protein	11.79
Rt679_8	adenylate cyclase	12.28
Rt782_64	ABC transporter substrate-binding protein	6.11
**Cell motility (N)**		
Rt651_28	chemotaxis protein	-9.59
Rt748_1	flagella associated protein	5,87
**Defense mechanisms (V)**		
Rt679_2	ABC transporter ATP-binding protein	11.76
Rt688_3	antibiotic resistance protein	7.05
Rt716_2	beta-lactamase	-5,61
Rt731_2	hemolysin D	6.63
**General function prediction only (R)**		
Rt651_16	amidohydrolase	-9.22
Rt659_29	oxidoreductase	-9.79
Rt704_2	hypothetical protein	-6.03
Rt759_1	3-phosphoglycerate dehydrogenase	5.96
**Function unknown (S)**		
Rt651_25	membrane protein	-8.19
Rt659_31	membrane protein	-9.07
**Mobilome (X)**		
Rt706_3	plasmid stabilization protein	6,54
Rt734_2	transposase	6.27
